# Theory of Sensation

**Published:** 1811-06

**Authors:** T. Smith

**Affiliations:** Bristol


### Medical and Physical Journal.

VOL. XXV.] JUNE, 1811. [NO. 148.

Printed for R. PHILLIPS, by E. Hemsted, Great New Street, Fetter Lane, London.

### *To the Editors of the Medical and Physical Journal.*

### *Theory of Sensation.*

GENTLEMEN,

AS Sensation is one of the most striking characteristics of animal life; and as painful or uneasy sensation forms a prominent feature in almost all diseases: so a knowledge of the real state of the living power and organ upon which it acts, under impressions that cause sensation, cannot fail to be highly interesting both in a medical and philosophical point of view.

In the following discussion an explanation of these has been attempted, which from the manner in which the enquiry has been conducted, will, it is hoped, not be found liable to any very material objections, care having been taken to avoid all hypotheses, and to advance nothing which is not either founded on universally received data, or supported by proofs derived from the unvariable phenomena of life.

Before offering a new explanation of the cause of Sensation, it may be proper to notice the opinions which have been already advanced. I shall do this as briefly as possible.

The only Theories of Sensation that I am acquainted with are the following. First, That it is caused by impressions made on the extremities of nerves, and communicated through them to the sensorium commune; and secondly, that it is truly an action of the sensorial power upon the fibres of the delicate vessels in which the nerves terminate, and that this action resembles muscular contraction.

Of the first of these opinions little requires to be said; it professes to teach us nothing of the state of the organ or part at which sensation is felt, under impressions; and seems to imply that the nerves are not endowed with vitality as well as the sensorium commune, a doctrine that is not proved, and certainly not altogether consonant to facts. The second opinion, that of Dr. Darwin, is more determinate; he has stated his doctrine clearly and explicitly, and has endeavoured to prove it by reference to facts. I do not know the general opinion of his Theory of Sensation; but if I am not mistaken, it is defective in this circumstance, that it does not inform us by what means the stimulus of light or other cause of Sensation induces the sensorial power to act on the fibres of the organ. This action of the vital power causing contraction of these fibres, certainly implies that previous thereto it had received some intelligence of the presence of the stimulus, and if so, it is quite superfluous to suppose contraction of fibres to be necessary to Sensation, since the Sensation must be already communicated before the action can take place.

In all philosophical investigations it ought never to be forgotten, not only that we are to admit no more causes of things than such as are both true and sufficient to explain the appearance, but that assigned causes, if true, ought always to produce the same effects in similar circumstances. Hence if a fibrous action, resembling the contraction of muscles, be in any instance not only a cause of Sensation but the Sensation itself, then I say that muscular contraction *ought in every instance to be felt;* otherwise the Theory is imperfect. But the contraction of muscles is in general not attended with Sensation in the fibres acting. In speaking we have no Sensation in the muscles which move the tongue. By attending to it we know, indeed, that our tongue moves, but that knowledge is derived from the Sensation produced in those parts of the mouth which the tongue touches, or in the tongue itself at those parts of it where contact with the teeth, gums, or palate takes place, and not in the fibres of the muscles that move that organ. When I *will* to lift my hand off this table I know that it moves obedient to volition, because I am informed of the motion by my sense of sight, or by my hand or arm touching some other part of my body, &c.; but I have no sensation in the muscles which acted in performing that intention of the will, and could not have known that these muscles did move if I had not been previously informed of the fact, or received conviction of it at the time, by applying my other hand and feeling a motion there. The same is equally true of all other muscles subject to the power of volition. The cause of any apparent deviations from this rule will, I trust, be explained to the entire satisfaction of the reader hereafter. With regard to the involuntary muscles, it must be obvious to every observer that the same want of Sensation obtains during their motions or actions. For in common cases we have no Sensation from the action on the heart and arteries, of the muscles of respiration, of the intestinal tinal canal, or from the motion of the lacteal and lymphatic systems; all of which perform their various functions without sensation in the power acting, or fibre acted upon.

From these facts I conclude that muscular contraction, although it may be in many, as it evidently is in some instances, an exciting cause, is not in one instance the sole cause of Sensation; and that some condition of the vital power and organic fibre, different from muscular contraction, must be present when Sensation is excited. What that condition is we will now endeavour to point out and demonstrate by the phenomena of Sensation.

In order to convey at once to the mind of my readers a clear idea of my views, I shall begin by stating what has appeared to me, after an attentive review of the phenomena of Sensation in health, and as far as my opportunities have permitted me, in diseases, to be the fundamental principle of Sensation; and then mention the phenomena in proof of that principle, which may be expressed as follows:

> Sensation is felt when an action attempted by the vital power is in any degree interrupted or obstructed: or in other words, when an action performed is less than the power exerted.

### *Proofs from the Phenomena.*

#### FIRST GENERAL PHENOMENON.

> When habitual or stimulated actions are interrupted by the abstraction or defect of those matters which are necessary to their continuance, a sensation is felt in the part where the interruption is; the efforts of the vital power to perform these actions continuing to be exerted.

#### FIRST INSTANCE.

A greater or less quantity of disengaged caloric* is essentially necessary to most, if not all, the actions of the living body. When more or less of that quantity is abstracted, it follows that a greater or less degree of interruption to the actions previously going on in the part from which it is taken must ensue. But when caloric is abstracted in unusual quan-

* I shall always use this word when the matter of heat is meant, to distinguish it from heat which I shall employ to express the Sensation only which caloric excites.

 tity, tity, an immediate Sensation of cold is felt. Hence it is evident this Sensation must be attended with interrupted action, and that the interruption is really the cause of the Sensation appears for the following reasons:

1st. When the actions interrupted are restored by the re-application of caloric, the Sensation of cold instantly ceases.

2d. When new actions (to which the abstraction of caloric is a stimulus) are successfully performed by the same power, and the former actions are discontinued, the Sensation ceases although the abstraction of caloric continues.

Thus, when a person enters the cold bath, he feels a very powerful Sensation of cold, and the temperature of his body falls. Commonly in a very short time the Sensation diminishes greatly, or goes entirely off, always as I have found by experiment before the temperature of the body arrives at the natural standard, but not till a progressive rise is indicated by the thermometer. In experiments of this kind, care ought always to be taken to distinguish between the Sensation of cold and the shivering which is sometimes a consequence of it: for in my experiments I have always found that the Sensation of cold was diminished by the shivering, and that when the shivering ceased before the temperature of the body had arrived at, or was rapidly ascending to the natural standard, the cold Sensation recurred with the effect of inducing a return of shivering. In general the temperature of the body rises gradually to the natural standard, which is a proof that the actions by which caloric is evolved are greater in the centre of the body at least, although the surface and extremities feel colder to another person—for the abstraction of caloric continuing greater than common, it must require an increased evolution of caloric to support the temperature. But since the actions of the surface to which caloric is necessary cannot be performed so easily while the greater abstraction continues, it is probable that the Sensation of cold ceasing or diminishing on the surface of the body is owing to the lesser exertions of the vital power on the surface, and greater in the centre or other parts of the system. A fact noticed by Dr. Currie (whose beautiful experiments related in the Medical Reports are well known) and which I myself have experienced, makes this amount almost to certainty: namely, that after the Sensation of cold had gone greatly or entirely off, and the temperature of the body was increasing; if the Sensation returned, a new fall of temperature speedily took place. This seems to show that the power which performed the actions on the surface that were interrupted by the abstraction of caloric on entering the bath, had really been employed in new actions in other parts of the system, while the sensation of cold was not not felt; and that an unsuccessful attempt to renew the interrupted actions produced the return of the coldness and diminished action where it was formerly increased, attended with a consequent fall of temperature.

3d. When the abstraction of caloric continues without any symptom of the interrupted action being restored, or the stimulated action successfully performed, the Sensation of cold is permanent.

Sometimes it happens that after going into the cold bath the temperature does not rise to the natural standard, but always keeps below: in these cases the cold Sensation continues. Sometimes librations of the temperature are observed keeping pace with the decrease and increase of the coldness. It may be said that the rise of temperature is the cause of the diminution of the coldness, and not the latter the cause of the former. To this I answer, not if the Sensation decreases before any sensible rise of temperature can be perceived—as I have found by experiment it does.

When the hands are exposed to a cold moist wind, they in general soon become somewhat swelled, and acquire a red or purple colour—symptoms which denote that the actions of the vessels of the part whether habitual or stimulated are retarded, a natural consequence of the greater accumulation of the fluids propelled into it by the circulation, as that accumulation is a natural consequence of the decreased action which the abstraction of caloric produced. Hence the Sensation of cold becomes more and more painful as the fluids accumulate and increase the resistance to the action of vessels. But increased resistance to action is interruption in degree. Therefore the general fact is proved as far as regards the abstraction or defect of caloric.

SECOND INSTANCE.

An occasional supply of food is as necessary to the unremitting continuation of the digestive actions of the stomach and lacteals, as a certain quantity of free caloric is to the vital actions in general. When the usual quantity of food has been taken into the stomach, it is certain this gives employment for a time at least to the whole powers of digestion. But in course of time this food is either absorbed by the lacteal vessels there, or carried forward into the small intestines, the stomach being again left empty, and the actions of the digestive powers of course interrupted—as certainly interrupted as the action of the heart and arteries would be if they were deprived of blood. Under this state of interrupted action of the organs of digestion, a Sensation of hunger is felt, evidently arising arising from that interruption, because it soon ceases after a new supply of food is taken into the stomach which permits them to return to unconstrained action free of sensation.

But sometimes the sensation of hunger goes off, if the supply of food has been withheld beyond the usual time. May not this depend on the same cause that produces a cessation of the coldness in the bath, although the abstraction of caloric continues the same, namely, an increase of action in other parts of the system? May not the action of the absorbents be increased in this case, and the living solids themselves be sometimes absorbed? Under privation of food with loss of appetite, is not there a more sudden loss of flesh and strength, than under similar privation when the appetite continues? It is extremely difficult to determine these questions in a manner that does not admit of some doubt. The following facts, however, the correctness of which may be depended on, seem to answer the last of these questions in the affirmative.

A person, with whom I am well acquainted, resolved to abstain entirely from food as long as he could, without any material inconvenience. He was in good health at the commencement of his abstinence. He became hungry at the usual time, but the hunger soon went off and did not return; but he was seized with head-ach, chilliness, great languor over his whole body and quick pulse. These symptoms were so much aggravated, and he became so feverish, that he thought it prudent, after persevering in his abstinence for four and twenty hours, to take a little food, although he had no appetite for it; and it is worthy of remark, that very soon after taking food, the headach ceased, the other symptoms abated, and in a few hours he felt himself perfectly recruited.

The same person at another time limited himself to the following diet. To breakfast he took about one ounce and an half of bread and one cup of tea. To dinner about the same quantity of bread, and one ounce of animal food; and in the evening as much bread with a cup of cold water. He persevered in this diet for three days, at the end of which he returned to his usual mode of eating. During these three days his appetite continued almost without intermission, the Sensation in his stomach becoming more painful every day. The food he took had very little effect in abating the hunger, which returned as keen as ever in less than a quarter of an hour after eating. The painful craving Sensation at his stomach was particularly troublesome after lying down in bed, and prevented sleep the greater part of the night. All this time he had neither headach, nor other uneasy sensation (that at his stomach excepted), his pulse seldom rose above

64 beats

64 beats in a minute, and, what is remarkable, he felt no perceptible diminution of his strength.

THIRD INSTANCE.

The circumstances attending the Sensation of thirst, seem to show that it arises from the interruption of the action of those vessels and glands which pour out and exhale the bland mucus which moistens the cavity of the mouth, fauces, stomach, &c. because thirst is felt when there is a total interruption to these actions known by dryness of the mouth, throat, &c. or when the mucus secreted is too viscid, which cannot but interrupt these actions in a greater or less degree. In either case the want of that fluidity which is necessary to these actions being duly performed, is the exciting cause of the Sensation, and the proximate cause (if I may be allowed the term) is interrupted action from that defect. The following circumstances are farther proofs, that thirst arises from the action of these vessels and glands being interrupted by defect of due fluidity. 1st. It often happens in cases of great thirst, that swallowing liquids has little or no effect in removing the sensation ; the cause of which is easily understood, when it is considered that the fluids which are necessary to exhalent action must be derived from the circulation ; of course a draught of water, unless it is immediately absorbed, and produces a determination of the fluids to these vessels, can have no immediate effect in removing the thirst. 2d. Dr. Currie particularly remarked that the cold affusion in fever was generally followed by a complete extinction of thirst ; which agrees with what would be expected to result from it, if interrupted action of the exhalents were the true cause ; for it is obvious that as the pabulum to these actions must be derived from the circulation, so nothing is better calculated or more likely to restore that, than determining the fluids copiously from the external to the internal parts. 3d. This is still further confirmed by what I have frequently observed in cases of fever attended with urgent thirst, which yielded to no kind of drink. Namely, that when a cathartic given operated with the effect of bringing off liquid evacuations, the thirst abated. But that the removal of the thirst was owing to the cathartic producing a greater determination of the fluids to the internal part, with a consequent renewal of the actions of the exhalent vessels, is obvious from this circumstance, that the sensation almost always went off before the medicine had produced any sensible evacuation. 4th. An emetic when it operates obviously produces an increased determination of the fluids to the internal parts, particularly the stomach stomach and mouth. But an emetic diminishes thirst, as appears from the great aversion which patients under the action of an emetic have to all kinds of drink. 5th. To a person that is thirsty nothing in general is more grateful than acid drinks, a small quantity of which often quenches thirst more effectually than a large quantity of water singly. But acids are well known to produce a greater flow of saliva, &c. and I suspect it is only in so far as they determine the fluids to the salival and mucous glands, and permit the interrupted actions to be renewed that they remove the sensation of thirst. 6th. Whatever greatly diminishes the quantity of the fluids in general, or much increases their viscidity, commonly causes a sensation of thirst; such as too copious evacuations of any kind, or an extreme evolution of caloric.

From these facts we may surely safely conclude that thirst arises from the actions of the exhalent vessels and glands of the mouth, fauces, stomach, &c. being interrupted by defect of the fluidity these actions require.

### FOURTH INSTANCE.

The habitual use of spirituous liquors, opium, tobacco, and other narcotic substances of the like kind, undoubtedly establishes certain actions, which require these substances to support them, in the same manner, I suppose, as food is required for the support of the digestive actions. A person who has accustomed himself to the use of snuff experiences an uneasy sensation when it is not applied as usual; and this Sensation goes instantly off when the snuff is applied, and again returns when the effect of this application is worn off. The same is true of all other acquired habitual actions.

### REMARKS.

Each of the Sensations belonging to this general phenomenon is peculiar to the exciting cause. Thus the abstraction of caloric is always attended with a Sensation of cold: the privation of food with hunger, the defect of fluidity with thirst, &c. but all of them have this in common, that they are accompanied with a desire of the peculiar substance, which is necessary to the renewal of the interrupted action; and they further agree in this, that the Sensation instantly ceases when the action is renewed by the necessary application: From which, and from the arguments before adduced, it is plain that uninterrupted action of the sensoreal power is not adequate to the production of sensation; and that the hypothesis which is built upon that supposition, instead of enabling abling us better to understand Sensation, only serves to bewilder our minds and render this subject, naturally intricate, still more perplexing and unintelligible. That action and sensation are truly opposite conditions of vitality appears from the instances above adduced ; and this view of the subject seems to render Sensation more intelligible, as it is not difficult to conceive, that an active intelligent power should *feel* when its motions are interrupted. In my next paper I shall resume the investigation of this principle, and hope I shall be able to prove, by reference to the other general phenomena, that Sensation in these also proceeds from interruption to action habitual or stimulated.

I shall conclude at this time, Gentlemen, by observing, that as I cannot help considering the idea here opened to be highly important, and I hope, useful in its consequences ; so I earnestly wish that any errors or oversights I have been or may be inadvertently guilty of in the prosecution of it, may not deter your intelligent readers from entering upon the examination of this interesting, though difficult subject, but rather stimulate them to assume the investigation, and supply the deficiencies of our knowledge by more able and diligent researches.

I am, respectfully, Gentlemen,
Your most obedient Servant,
T. SMITH.

*Bristol, April 10th,* 1811.

